# From Waste Paper to Regenerated Nanocellulose: Purification Strategy Controls the Residual Chemical Fingerprint

**DOI:** 10.3390/polym18182242

**Published:** 2026-09-15

**Authors:** Helena Raclavská, Barbora Švédová, Michal Šafář, Marek Kucbel, Jana Růžičková, Dalibor Matýsek, Karolina Slamová, Pavel Kantor

**Affiliations:** 1 ENET Centre, CEET, VSB–Technical University of Ostrava, 17. Listopadu 15/2172, 708 00 Ostrava, Czech Republic; helena.raclavska@vsb.cz (H.R.); barbora.svedova@vsb.cz (B.Š.); michal.safar@vsb.cz (M.Š.); jana.ruzickova@vsb.cz (J.R.); pavel.kantor@vsb.cz (P.K.); 2 Department of Geological Engineering, Faculty of Mining and Geology, VSB–Technical University of Ostrava, 17. Listopadu 15/2172, 708 00 Ostrava, Czech Republic; dalibor.matysek@vsb.cz; 3 Institute of Foreign Languages, VSB–Technical University of Ostrava, 17. Listopadu 15/2172, 708 00 Ostrava, Czech Republic; karolina.slamova@vsb.cz

**Keywords:** waste paper, regenerated nanocellulose, dissolution–regeneration, Py-GC/MS, residual organic compounds, application-oriented purification

## Abstract

Waste paper is a promising secondary cellulose resource for nanocellulose production, but residual constituents originating from its previous manufacturing and recycling history may affect the chemical quality of the final material. This study evaluated purification strategies for regenerated nanocellulose (RNC) produced from waste office paper using X-ray diffraction (XRD), attenuated total reflectance Fourier-transform infrared spectroscopy (ATR-FTIR), and pyrolysis–gas chromatography/mass spectrometry (Py-GC/MS), with commercial cellulose nanocrystals (CNCs) as a reference. Direct dissolution–regeneration retained substantial concentrations of residual non-cellulosic organic compounds (542.2 mg·kg^−1^). It generated a membrane-associated fraction representing 18% of the recovered dry mass but containing 53% of the quantified residual organic load. Post-regeneration hexane–ethanol purification reduced the organic residuum to 11.9 mg·kg^−1^, whereas acetic acid pretreatment followed by dissolution–regeneration resulted in 20.0 mg·kg^−1^ and prevented formation of the membrane-associated fraction. Both values were substantially below that of commercial CNCs (229.1 mg·kg^−1^). The results demonstrate that the purification strategy controls both residual chemical composition and fractionation behaviour during processing, supporting an application-oriented purification concept in which purification intensity is matched to the requirements of the intended nanocellulose application.

## 1. Introduction

The increasing demand for sustainable materials and the transition toward a circular economy have intensified interest in the high-value conversion of cellulose-rich waste streams. Waste paper represents an attractive secondary cellulose resource because of its wide availability, low cost, and relatively low lignin and hemicellulose contents compared with primary lignocellulosic biomass [1]. Targeted fractionation can also enable recovery of nanocellulose alongside valuable non-cellulosic fractions, supporting integrated valorisation of secondary biomass resources [2]. Recovered paper has consequently been investigated as a feedstock for cellulose nanocrystals (CNCs), cellulose nanofibers (CNFs), regenerated nanocellulose, and related materials [3,4]. Successful conversion of office and mixed waste paper has been demonstrated for barrier coatings, membranes, adhesives, and composites, while laser-printed office paper has also been identified as a viable nanocellulose feedstock [5,6,7].

Waste paper, however, is not simply a cellulose-rich feedstock. Paper products contain mineral fillers, sizing agents, binders, printing inks, coatings, adhesives, plasticisers, waxes, and other functional additives. At the same time, repeated recycling may promote the transfer and accumulation of constituents from different paper grades and associated materials [8]. Chemical profiling of recovered paper has revealed diverse organic compounds related to papermaking, printing, adhesives, polymeric materials, and previous product use [9], while sticky contaminants further contribute to the heterogeneity of recycled fibre streams [10]. In our previous study, precipitated calcium carbonate (PCC) was identified as the dominant mineral filler in waste office paper, and treatment with 0.2 M acetic acid removed 86% of PCC while simultaneously reducing paper- and printing-associated organic compounds [11]. However, part of the organic residuum remained associated with the recovered cellulose. Conversion of waste paper into nanocellulose, therefore, involves not only structural transformation of cellulose but also the removal, redistribution, transformation, or persistence of constituents inherited from the previous lifecycle of the feedstock.

Several routes are available for nanocellulose production from recovered paper. CNCs are typically obtained by acid hydrolysis, whereas CNFs are predominantly produced by mechanical fibrillation, often assisted by chemical or oxidative pretreatments [12,13]. Material characteristics depend on both feedstock and processing route [14], while production costs, energy consumption, and process complexity remain important constraints on industrial implementation [15,16]. Alternatively, cellulose can be dissolved in low-temperature non-derivatising NaOH-based systems containing urea and/or thiourea and subsequently regenerated [17,18]. Regeneration results in reassociation of cellulose chains into a new supramolecular organisation, frequently accompanied by the transformation of Cellulose I into Cellulose II [19]. Dissolution–regeneration thus represents a fundamentally different route from conventional CNC or CNF production.

Although numerous studies have demonstrated the feasibility of producing nanocellulose from waste paper, most have focused on production efficiency and the morphological, structural, thermal, or mechanical properties of the resulting materials [1,13]. Conversely, studies of paper recycling have documented the complex mixture of additives, polymer-derived residues, sticky contaminants, and other organic constituents present in recovered fibre streams [9,10]. These two aspects have rarely been integrated, and the fate of the chemical residuum inherited from recycled paper during nanocellulose production remains poorly understood. In particular, it is unclear whether paper-associated organic compounds are removed during dissolution–regeneration, retained in the regenerated cellulose, or selectively redistributed between processing fractions. Structural and morphological characterisation alone cannot address this gap. Moreover, because the required degree of purification may depend on the intended application, the relationship between processing route and residual chemical composition is important for rational valorisation of secondary cellulose resources [15,16].

The present study addresses this gap by integrating structural characterisation with compound-resolved analysis of the residual non-cellulosic organic residuum during conversion of waste office paper into regenerated nanocellulose. Direct dissolution–regeneration followed by post-regeneration solvent purification was compared with upstream acetic acid pretreatment followed by dissolution–regeneration. XRD and ATR-FTIR were used to assess cellulose structural reorganisation, while Py-GC/MS tracked residual organic constituents and their redistribution during processing; commercial CNCs served as an independent reference. This approach enables evaluation of purification strategies in terms of both cellulose structure and the persistence, fractionation, and removal of the chemical legacy inherited from recycled paper, providing a basis for application-oriented purification of waste paper-derived regenerated nanocellulose.

## 2. Materials and Methods

### 2.1. Waste Office Paper and Reference Material

Waste office paper was used as the secondary cellulose feedstock. The material was obtained from a commercial paper-shredding facility in Ostrava, Czech Republic, and originated from the waste office paper previously characterised by Kucbel et al. [11], who provided details of its origin, sampling, and initial chemical characterisation. Commercial cellulose nanocrystals (CNCs; Nanografi, Ankara, Turkey; product code NG01NC0101) were included as a reference material to provide a benchmark for comparison of the structural (XRD and FTIR) characteristics and the organic residuum determined by Py-GC/MS. Because the feedstocks and manufacturing routes are fundamentally different, the commercial CNC and the regenerated nanocellulose derived from recycled office paper were not intended to represent directly equivalent materials. Consequently, the comparison was performed to provide a structural and chemical context rather than to evaluate overall material quality or production efficiency.

### 2.2. Experimental Design and Acetic Acid Pretreatment

**Figure 1:**
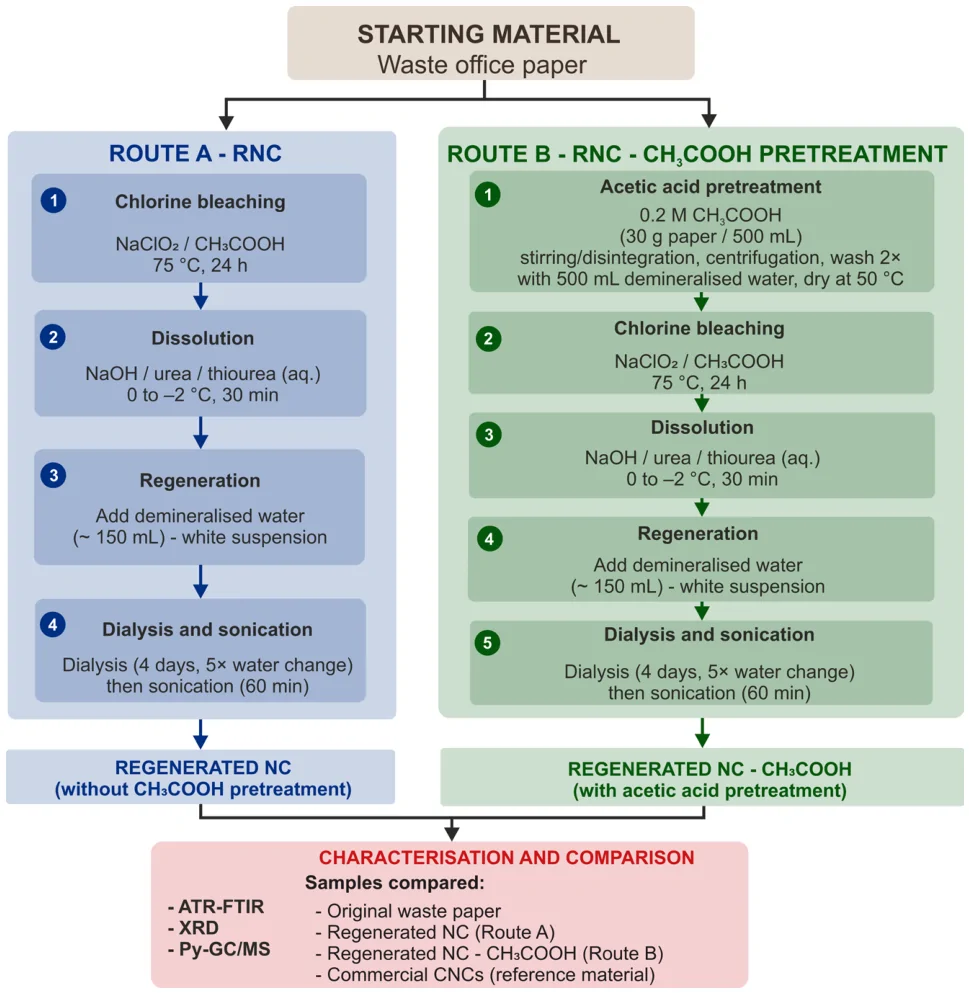
Experimental design for the preparation and comparative characterisation of regenerated nanocellulose from waste office paper previously characterised by Kucbel et al. [11]. Route A (blue) represents the modified Zhang et al. [20] procedure applied directly to waste office paper, whereas Route B (green) represents the modified Zhang et al. [20] procedure incorporates 0.2 M CH_3_COOH pretreatment based on Kucbel et al. [11] before the same dissolution–regeneration procedure.

Two parallel processing routes were applied to the same waste office paper feedstock (Figure 1). In Route A, waste office paper was processed directly using a modified low-temperature dissolution–regeneration procedure based on Zhang et al. [20], without prior PCC removal. In Route B, the same material was first subjected to the acetic acid (Merck KGaA, Darmstadt, Germany) pretreatment developed by Kucbel et al. [11], followed by the same modified Zhang et al. [20]. procedure. For Route B, 30 g of waste office paper was disintegrated in 500 mL of 0.2 M acetic acid using a Vorwerk Thermomix TM6 (Vorwerk Elektrowerke GmbH & Co. KG, Wuppertal, Germany). The suspension was centrifuged using an Avanti J-E centrifuge (Beckman Coulter, Inc., Brea, CA, USA), and the recovered solid fraction was washed twice with 500 mL of demineralised water and dried at 50 °C in a drying oven (Memmert GmbH & Co. KG, Schwabach, Germany). This pretreatment was previously shown to remove a substantial proportion of PCC and reduce the associated organic chemical load [11].

### 2.3. Bleaching, Dissolution, and Regeneration of Cellulose

Both processing routes followed the same modified low-temperature dissolution–regeneration procedure based on Zhang et al. [20], as summarised in Figure 1. For each route, 13 g of material was dispersed in 200 mL of demineralised water using a Vorwerk Thermomix TM6. The suspension was transferred to a 400 mL beaker, supplemented with 2 mL of acetic acid and 31.2 mL of 25% NaClO_2_ solution (Merck KGaA, Darmstadt, Germany), and adjusted with demineralised water to a final volume of 260 mL. The beaker was covered with a watch glass and maintained at 75 °C for 24 h. The bleached fibres were washed with demineralised water by filtration until a neutral pH was reached, then washed with acetone (Merck KGaA, Darmstadt, Germany) and dried.

A low-temperature aqueous solvent was prepared by dissolving 8.0 g of NaOH (Merck KGaA, Darmstadt, Germany), 8.0 g of urea (Merck KGaA, Darmstadt, Germany), and 6.5 g of thiourea (Merck KGaA, Darmstadt, Germany) in demineralised water to a final volume of 100 mL and pre-cooled to −10 °C. For each route, 5.0 g of dried bleached material was mixed with 100 mL of the pre-cooled solution and maintained at 0 to −2 °C under continuous mixing for 30 min. The undissolved fraction was removed by centrifugation at 4800 rpm for 30 min.

The resulting transparent cellulose-containing supernatant was transferred to a beaker, and approximately 150 mL of demineralised water was gradually added to induce cellulose regeneration. The resulting materials were designated RNC-Zhang et al. [20] (Route A) and RNC-CH_3_COOH-Zhang et al. [20] (Route B) and subjected to the same dialysis and sonication procedure.

### 2.4. Dialysis and Sonication

Following regeneration, the RNC-Zhang et al. [20] (Route A) and RNC-CH_3_COOH-Zhang et al. [20] (Route B) suspensions were purified using the same dialysis procedure. Approximately 70 mL of each suspension was transferred into a dialysis membrane Spectra/Por^®^ 7 pre-treated regenerated cellulose dialysis tubing (molecular weight cut-off, MWCO: 1 kDa; product code 132105; Spectrum Laboratories, Inc., Rancho Dominguez, CA, USA) and immersed in approximately 10 L of demineralised water. The dialysis water was replaced twice on the first day and once daily on the second and third days, followed by a final replacement on the fourth day, for a total of five water exchanges over four days.

After dialysis, the suspensions were sonicated in a Sonorex RK 52 ultrasonic bath (Bandelin electronic GmbH & Co. KG, Berlin, Germany) for 60 min to disrupt aggregates and obtain stable dispersions of regenerated nanocellulose. A portion of each suspension was dried at 40–50 °C for further characterisation, while the remainder was retained as an aqueous suspension.

For the direct dissolution–regeneration route, two physically distinguishable solid fractions were recovered during dialysis: the RNC suspension and a membrane-associated fraction. After drying, these represented approximately 82% and 18%, respectively, of their combined recovered dry mass. No comparable membrane-associated fraction was observed during dialysis of CH_3_COOH-pretreated cellulose subjected to the same dissolution–regeneration procedure.

### 2.5. Additional Solvent Purification of Regenerated Nanocellulose

An additional solvent-cleaning step was performed to evaluate the removal of residual organic compounds from regenerated nanocellulose. The suspension was centrifuged, the aqueous supernatant discarded, and hexane (Merck KGaA, Darmstadt, Germany) added at approximately 10–15 times the volume of the recovered material. The closed vessel was mixed for 30 min, centrifuged, and the hexane phase was removed. The same procedure was subsequently performed with ethanol (Merck KGaA, Darmstadt, Germany). This sequential extraction cycle was repeated once, resulting in the sequence hexane–ethanol–hexane–ethanol. The nanocellulose was then washed two to three times with demineralised water, with centrifugation after each washing step, and dried at 40–50 °C.

### 2.6. Characterisation of Regenerated Nanocellulose

#### 2.6.1. ATR-FTIR Spectroscopy

ATR-FTIR spectra were recorded using a Nicolet 6700 FTIR spectrometer (Thermo Scientific, Waltham, MA, USA) equipped with an ATR accessory over 4000–525 cm^−1^ at a resolution of 4 cm^−1^, with 64 scans per sample. Spectra were evaluated for characteristic cellulose absorption bands and changes associated with structural reorganisation.

#### 2.6.2. X-Ray Diffraction (XRD)

XRD analysis was performed using a Bruker AXS D8 Advance diffractometer (Bruker AXS GmbH, Karlsruhe, Germany) in coupled θ/2θ geometry with CuKα radiation (λ = 1.54060 Å). The instrument was equipped with a Göbel mirror in the primary beam and a position-sensitive LynxEye semiconductor detector (Bruker, Billerica, MA, USA). Diffraction patterns were used to evaluate changes in the supramolecular organisation of cellulose and to compare it with commercial CNCs. The crystallinity index (CrI) was calculated from the XRD patterns to provide a relative comparison of crystalline ordering. For regenerated nanocellulose, which exhibited Cellulose II-like diffraction features, CrI was determined using the modified Segal approach based on the intensity of the principal (020) reflection and the amorphous intensity at approximately 16° 2θ, following the methodology proposed by Nam et al. [21] and subsequently applied to regenerated cellulose nanoparticles by Campuzano et al. [22]. Because the Segal method is an empirical peak-height approach, the resulting CrI values were interpreted as relative indicators of crystalline ordering rather than absolute crystallinity.

#### 2.6.3. Pyrolysis–Gas Chromatography/Mass Spectrometry (Py-GC/MS)

Py-GC/MS was used to evaluate the persistence and redistribution of organic constituents during processing. Approximately 100–500 µg of each sample was placed in a metal sample cup together with 1,3,5-tri-tert-butylbenzene (Merck KGaA, Darmstadt, Germany) as an internal standard. Pyrolysis was performed in single-shot mode at 600 °C for 60 s using a Frontier Lab AS2020E (Frontier Laboratories Ltd., Koriyama, Fukushima, Japan) microfurnace pyrolysis system coupled to an Agilent 7890/5977C GC/MS system (Agilent Technologies, Inc., Santa Clara, CA, USA). Pyrolysates were introduced into the GC system at 300 °C and separated on an Ultra ALLOY^®^ UA+Py2 (Frontier Laboratories Ltd., Koriyama, Fukushima, Japan) capillary column (30 m × 0.25 mm × 0.50 µm). Compounds were identified by comparison of acquired mass spectra with F-Search version 3.8 (Frontier Laboratories Ltd., Koriyama, Fukushima, Japan) and the National Institute of Standards and Technology (NIST) mass spectral libraries (Gaithersburg, MD, USA).

The identified compounds were evaluated semi-quantitatively relative to the internal standard, and abundances were expressed as concentration equivalents in mg·kg^−1^ of dry material. This approach was used to compare processing routes and purification stages rather than for compound-specific absolute quantification. All samples were analysed in duplicate. Concentrations reported in Table S1 represent arithmetic means of the two replicate analyses, while individual replicate-level data are available in the associated Zenodo dataset.

To preserve traceability while standardising nomenclature, Table S1 reports the original mass-spectral-library name together with a standardised compound name and, where an unambiguous structure could be assigned, the corresponding systematic International Union of Pure and Applied Chemistry (IUPAC) name. No IUPAC name was assigned to unresolved or group-level library identifications.

The experimentally determined dry-mass proportions of the RNC and membrane-associated fractions (82:18) were used to calculate the relative mass distribution of individual residual non-cellulosic compounds between the two recovered solid fractions based on their Py-GC/MS concentrations and corresponding dry-mass proportions. These calculations do not constitute a complete process mass balance because compounds present in the dialysis medium and other process losses were not quantified.

### 2.7. Data Processing and Chemical Classification

For interpretation of the Py-GC/MS profiles, cellulose-derived pyrolysis products, particularly anhydrosugars and other carbohydrate-derived compounds, were distinguished from residual non-cellulosic organic constituents and excluded from the assessment of the residual organic fingerprint.

Residual compounds were classified as waxy/polyolefinic hydrocarbons; plasticisers and polymer additives; aromatic and condensed aromatic compounds; lipid-derived, wax-like and oxygenated degradation products; amide additives and aromatic amides; terpenoid and biologically associated hydrophobic products; process-related nitrogen- and sulphur-containing compounds; oxygenated transformation products; oxidised and secondary transformation products; and NIAS (non-intentionally added substances) and polymer-derived transformation products. The same classification was applied to all samples, including commercial CNCs. Concentrations of individual compounds within each group were summed to obtain group-level semiquantitative abundances for comparison among processing routes, purification stages, and the commercial CNC reference.

## 3. Results

### 3.1. Structural Reorganisation of Regenerated Nanocellulose

**Figure 2:**
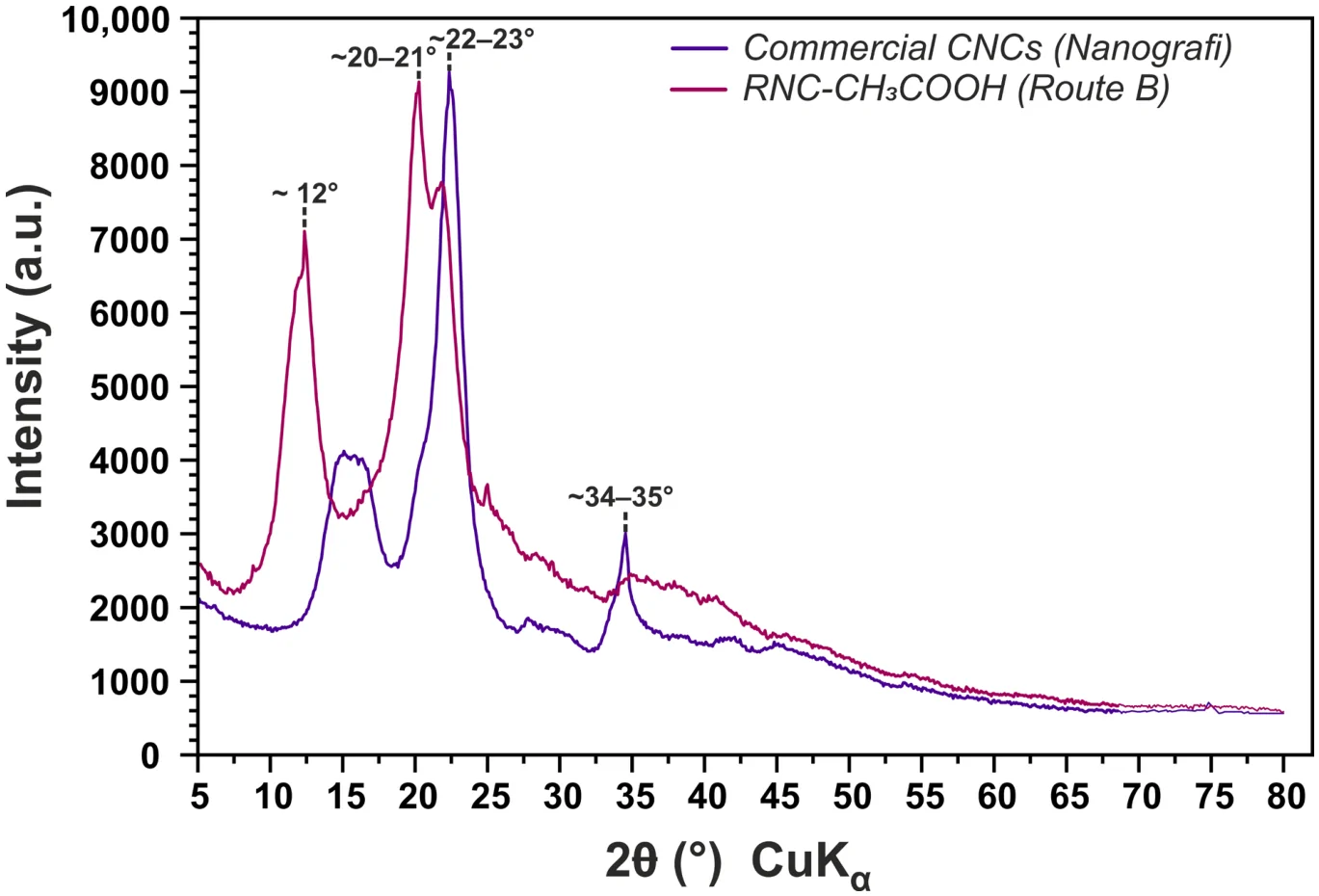
X-ray diffraction patterns of regenerated nanocellulose prepared from acetic acid-pretreated waste office paper using the modified Zhang et al. [20]) procedure (RNC-CH_3_COOH-Zhang et al. [20]; Route B) and commercial cellulose nanocrystals (CNCs). Intensities are presented in arbitrary units to facilitate a comparison of the diffraction profiles.

X-ray diffraction revealed pronounced differences in the supramolecular organisation of waste paper-derived regenerated nanocellulose (RNC) and the commercial CNC reference (Figure 2). Commercial CNCs exhibited a relatively sharp reflection at approximately 22–23° 2θ, consistent with a highly ordered cellulose structure. In contrast, RNC prepared from acetic acid-pretreated waste office paper exhibited a pronounced reflection at approximately 12° 2θ together with a broader and redistributed diffraction profile within the 20–23° 2θ region.

These changes are consistent with structural reorganisation during cellulose dissolution and regeneration. Dissolution disrupts the intermolecular interactions and hydrogen-bonding network of native cellulose, whereas regeneration allows cellulose chains to reassociate into a new supramolecular structure [23,24]. Analogous dissolution–regeneration approaches have been used to prepare regenerated cellulose nanoparticles and nanofibers, supporting the designation regenerated nanocellulose (RNC) for nanostructured cellulose obtained through this processing route [25,26].

The reflection at approximately 12° 2θ is particularly indicative of this structural transformation. Cellulose regenerated after dissolution has been reported to exhibit Cellulose II-related reflections at approximately 12°, 20°, and 22° 2θ [26], and transformation from Cellulose I into Cellulose II has also been demonstrated specifically for cellulose processed in aqueous NaOH/urea systems [27]. The diffraction pattern observed here therefore indicates the development of Cellulose II-like structural organisation during regeneration. This interpretation is consistent with the disruption and subsequent reorganisation of the cellulose hydrogen-bonding network in alkaline aqueous systems [24]. Urea contributes to the stabilisation of dissolved cellulose within the cellulose–alkali–water environment, with recent nuclear magnetic resonance (NMR) evidence providing further molecular-level insight into its role in cellulose dissolution [28].

However, the RNC reflections were broad and partially overlapping, and quantitative phase analysis was not performed. Therefore, the data do not support the assignment of the material as a completely transformed Cellulose II but rather indicate substantial supramolecular reorganisation with the development of Cellulose II-like features. The broader diffraction profile relative to commercial CNCs additionally indicates lower long-range structural ordering, consistent with the different formation mechanisms of the two materials. Conventional CNC production preferentially retains highly ordered cellulose domains, whereas dissolution–regeneration disrupts the pre-existing supramolecular organisation before cellulose chains reassociate into a regenerated structure [26].

To complement the qualitative interpretation of the XRD patterns, the crystallinity index (CrI) was determined for the commercial CNC reference material and the regenerated nanocellulose prepared after CH_3_COOH pretreatment (Table 1). Commercial CNC exhibited a higher CrI (70.6%) than regenerated nanocellulose (61.6%), consistent with the higher structural order expected for highly crystalline cellulose nanocrystals. The regenerated nanocellulose nevertheless retained a relatively high CrI despite the development of Cellulose II-like structural features during dissolution–regeneration. This interpretation is consistent with previous studies applying the modified Segal approach to Cellulose II and regenerated cellulose materials [21,22]. Because the modified Segal method provides an empirical index of relative crystalline ordering rather than an absolute measure of crystallinity, the obtained CrI values were used only for comparative interpretation of the investigated materials.

In summary, XRD confirmed that low-temperature dissolution–regeneration substantially reorganised the cellulose structure recovered from waste office paper. The regenerated nanocellulose exhibited characteristic Cellulose II-like structural features while retaining a relatively high degree of crystalline ordering despite extensive structural reorganisation.

#### 3.1.1. ATR-FTIR Spectroscopy and FTIR-Derived Structural Indices

ATR-FTIR spectroscopy confirmed that all regenerated nanocellulose (RNC) variants retained the characteristic cellulose framework irrespective of the applied purification route (Table 2). RNC-Zhang et al. [20] (Route A), RNC-Zhang et al. [20]-solvent, and RNC-CH_3_COOH-Zhang et al. [20] (Route B) exhibited the characteristic absorption bands of cellulose (Table 1), with broad O–H stretching at approximately 3600–3000 cm^−1^, C–H and CH_2_ stretching at 2940–2840 cm^−1^, CH_2_ bending near 1430 cm^−1^, C–O–C vibrations at approximately 1135–1180 cm^−1^, C–O stretching around 1050 cm^−1^, and β-glycosidic linkages near 895–900 cm^−1^ [29]. The persistence of these characteristic bands indicates that neither acetic acid pretreatment nor post-regeneration solvent purification caused major chemical modification of the cellulose backbone. This is particularly relevant for the acetic acid pretreatment, which previously removed 86% of the PCC mineral fraction from the same waste-paper feedstock [11].

To complement the qualitative interpretation of the ATR-FTIR data, the lateral order index (LOI; A_1420_/A_895_) and total crystallinity index (TCI; A_1372_/A_2900_) were calculated (Table 3). Commercial CNCs exhibited the highest LOI (2.29), followed by RNC-Zhang et al. [20] (Route A) (1.93), RNC-Zhang et al. [20]-solvent (1.50), and RNC-CH_3_COOH-Zhang et al. [20] (Route B) (0.95). In contrast, TCI did not follow the same trend, with values of 0.40, 0.74, 0.53, and 1.17, respectively. LOI and TCI are empirical spectral ratios sensitive to cellulose molecular organisation, hydrogen bonding, conformation, and polymorphic structure and should not be interpreted as direct quantitative measures of crystallinity [30,31].

The contrasting behaviour of the two indices was most evident for RNC-CH_3_COOH-Zhang et al. [20] (Route B), which exhibited the lowest LOI but the highest TCI. This divergence shows that LOI and TCI respond differently to structural changes induced by pretreatment and dissolution–regeneration. It highlights the limitations of assigning crystallinity based on individual FTIR band ratios. The pronounced decrease in LOI nevertheless suggests modification of local cellulose organisation, consistent with the structural reorganisation independently observed by XRD. Accordingly, conclusions regarding supramolecular organisation were based primarily on XRD, while LOI and TCI were treated as complementary structural indicators [31].

Post-regeneration hexane/ethanol purification of RNC-Zhang et al. [20] (Route A) did not result in the disappearance or formation of major cellulose absorption bands, indicating that solvent purification primarily affected extractable constituents while preserving the cellulose matrix. In summary, ATR-FTIR results confirm preservation of the fundamental cellulose framework across the investigated purification routes, while the FTIR-derived indices indicate differences in local molecular organisation.

#### 3.1.2. Dry Matter Content and Material Yield

The regenerated nanocellulose suspensions obtained after dissolution–regeneration exhibited dry matter concentrations of 13.7 g·L^−1^ for the untreated waste-paper feedstock and 10.0 g·L^−1^ after acetic acid pretreatment. Based on the final suspension volume of 250 mL, this corresponds to the recovery of 3.43 g and 2.50 g of regenerated nanocellulose from an initial 5 g of waste office paper, equivalent to approximately 685 g·kg^−1^ and 500 g·kg^−1^ of the original feedstock, respectively (Table 4).

The approximately 27% reduction in recovered dry mass after acetic acid pretreatment should not be interpreted as cellulose loss alone, but primarily as the intentional removal of precipitated calcium carbonate (PCC) and other non-cellulosic constituents before dissolution–regeneration. Consequently, although the pretreatment decreases the total recovered dry mass, it simultaneously produces regenerated nanocellulose with a substantially lower organic residuum, as demonstrated by the subsequent Py-GC/MS analysis. This interpretation is further supported by the marked reduction in mineral filler content observed after pretreatment and by the significantly lower concentrations of residual organic compounds in the final regenerated nanocellulose.

The lower recovered dry mass observed after acetic acid pretreatment should not be interpreted solely as cellulose loss. Instead, it reflects the combined effects of selective removal of acid-soluble non-cellulosic constituents, including mineral fillers, together with unavoidable material losses during washing, centrifugation, and dissolution–regeneration. Consequently, the recovered dry mass cannot be directly equated with cellulose recovery, and the present data do not permit quantitative separation of cellulose loss from the removal of non-cellulosic components.

### 3.2. Fate of Residual Organic Compounds During Nanocellulose Production

Py-GC/MS analysis revealed a chemically complex profile comprising both cellulose-derived pyrolysis products and compounds associated with non-cellulosic constituents of the waste-paper feedstock. Characteristic carbohydrate-derived products, including anhydrosugars, furans, and other oxygenated compounds, were also prominent in commercial CNCs and were, therefore, considered markers of the cellulose matrix rather than residual chemical contamination. Consequently, cellulose-derived pyrolysis products were excluded from the assessment, which focused exclusively on the residual non-cellulosic organic fingerprint determined by Py-GC/MS. The compounds included in this evaluation, together with their nomenclature, classification, interpretative assignment, and concentrations in individual processing fractions, are reported in Table S1.

This fingerprint provides information complementary to XRD and ATR-FTIR by specifically addressing non-cellulosic organic constituents retained, transformed, or redistributed during processing. Such characterisation is relevant because extractable and leachable impurities represent an important aspect of the chemical assessment of highly purified nanocellulosic materials [32]. The evaluated fingerprint included persistent paper-associated residues originating from manufacture, converting, printing, use, and recycling, as well as transformation products of non-cellulosic paper constituents. Recycled paper may contain compounds derived from inks, coatings, adhesives, sizing agents, polymeric materials, mineral-oil formulations, and plasticisers that may persist through successive recycling cycles [33,34,35,36].

Because individual compounds cannot always be assigned unequivocally to a single technological source, interpretation was based on chemical structure together with probable material origin. Residual lignin-associated compounds were retained because lignin represents a non-target constituent of RNC. At the same time, aromatic compounds were not interpreted exclusively as lignin-derived because they may also originate from inks, coatings, adhesives, polymeric materials, or secondary thermal transformations. Long-chain *n*-alkanes, including hexacosane, octacosane, and nonacosane, were similarly interpreted as persistent wax- or hydrocarbon-associated residues rather than cellulose-derived pyrolysis products, consistent with their potential occurrence in waxes, coatings, printing-related materials, and other hydrocarbon-containing formulations [37,38]. Residual compounds were classified into waxy/polyolefinic hydrocarbons, plasticisers and polymer additives, aromatic and condensed aromatic compounds, lipid-derived, wax-like and oxygenated degradation products, amide additives and aromatic amides, terpenoid and biologically associated hydrophobic products, process-related nitrogen- and sulphur-containing compounds, oxygenated transformation products, oxidised and secondary transformation products, and NIAS and polymer-derived transformation products. Their subsequent evaluation focused on whether these residual constituents were retained, redistributed between processing fractions, or removed during the investigated purification and dissolution–regeneration routes. Acetic acid pretreatment of the same waste-paper feedstock had previously been shown to substantially reduce both the PCC mineral fraction and part of its associated organic chemical load [11].

#### 3.2.1. Differential Distribution of Paper-Derived Organic Compounds During Dissolution–Regeneration

**Figure 3:**
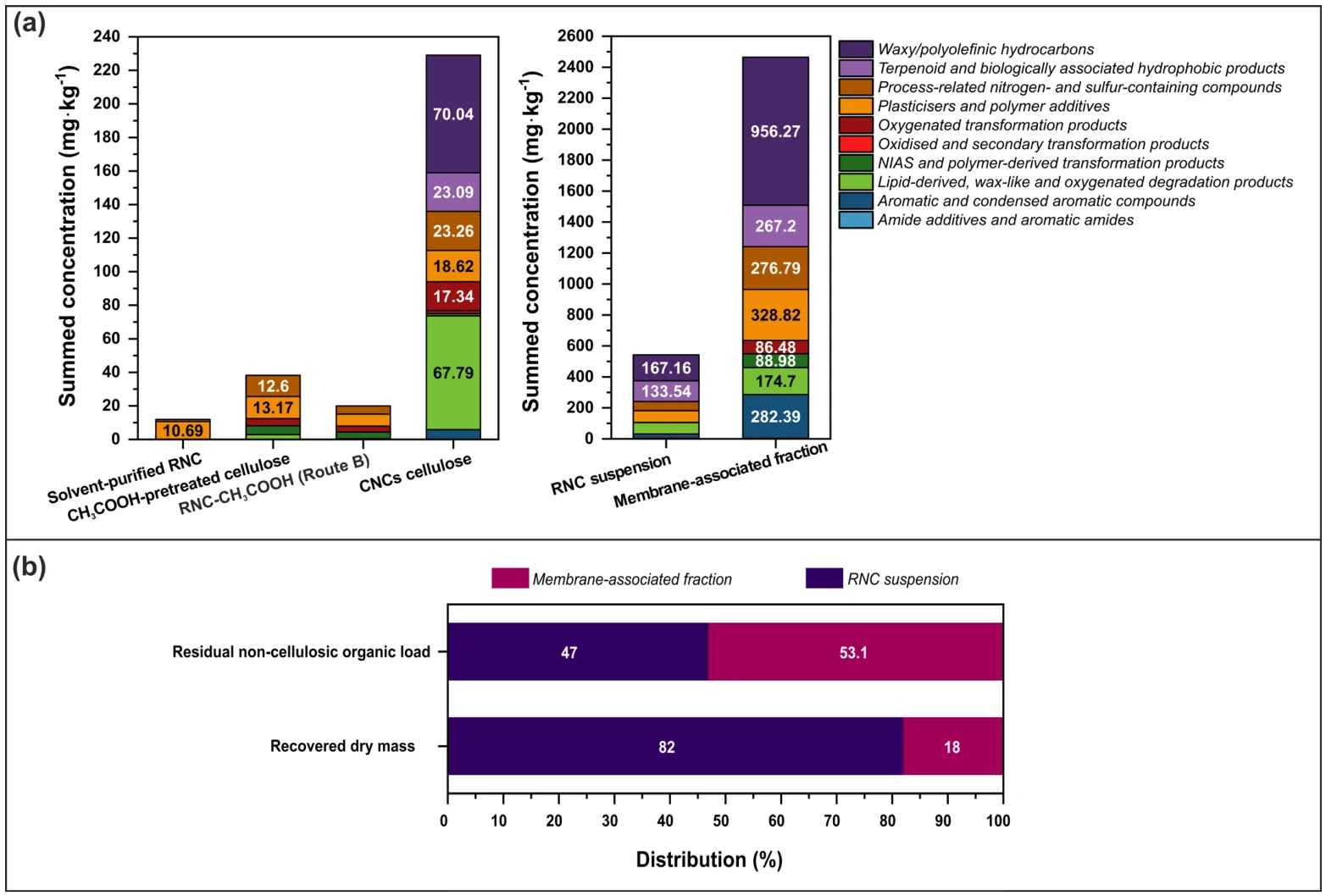
Residual non-cellulosic organic compounds during direct dissolution–regeneration of waste office paper. (**a**) Summed concentrations of compound groups determined by Py-GC/MS. The left plot compares solvent-purified RNC, CH_3_COOH-pre-treated cellulose, RNC–CH_3_COOH (Route B, prepared using the modified Zhang et al. [20] procedure), and commercial CNCs. The right plot compares the RNC suspension and membrane-associated fraction recovered during direct dissolution–regeneration (Route A). (**b**) Distribution of recovered dry mass and quantified residual non-cellulosic organic load between the two Route A fractions, calculated using their experimentally determined dry-mass proportions (82:18). Individual compounds are reported in Table S1.

Direct dissolution–regeneration of waste office paper did not eliminate its non-cellulosic organic residuum. The RNC suspension retained several groups of paper-associated compounds, dominated by waxy/polyolefinic hydrocarbons, terpenoid and biologically associated hydrophobic products, plasticisers and polymer additives, and aromatic and condensed aromatic compounds (Figure 3a). This persistence is consistent with the complex composition of recycled paper, in which constituents originating from printing, coatings, adhesives, waxes, and polymeric additives may remain associated with the fibre matrix during recycling and subsequent processing [9,33].

A substantially different profile was observed in the membrane-associated fraction formed during dialysis, where concentrations of most major hydrophobic compound groups were considerably higher than in the RNC suspension (Figure 3a). Waxy/polyolefinic hydrocarbons dominated, accompanied by plasticisers and polymer additives, aromatic and condensed aromatic compounds, lipid-derived, wax-like and oxygenated degradation products, terpenoid and biologically associated hydrophobic products, and process-related nitrogen- and sulphur-containing compounds. The membrane-associated material (Figure 3b), therefore, represented a chemically distinct fraction enriched in residual non-cellulosic constituents.

Importantly, this enrichment was not limited to concentration differences. The membrane-associated material represented approximately 18% of the combined recovered dry mass, compared with 82% for the RNC suspension (Figure 3b). After normalisation to the experimentally determined dry-mass proportions (82:18), the membrane-associated fraction accounted for 53.1% of the quantified residual non-cellulosic organic load recovered in the two solid fractions after dialysis, compared with 46.9% in the RNC suspension. Organic compounds transferred to the dialysis medium were not quantified and are, therefore, not included in this distribution. Thus, a relatively small fraction of the recovered material carried a disproportionately large share of the residual organic load, demonstrating pronounced chemical fractionation during dissolution–regeneration and subsequent dialysis.

Preferential distribution was particularly pronounced for several persistent compounds. Approximately 59.4% of hexacosane and 97.7% of nonacosane present in the two recovered fractions were associated with the membrane-associated material (Table S1). These higher *n*-alkanes were interpreted as persistent wax- or hydrocarbon-associated residues rather than cellulose-derived pyrolysis products. They may originate from paraffinic waxes, coatings, printing-related materials, and other hydrophobic formulations associated with paper and packaging [37,38]. Similarly, 75.7% of the bis(2-ethylhexyl) phthalate (DEHP) mass was associated with the membrane-associated fraction. This behaviour is consistent with hydrophobic stickies and deposit-forming materials in recycled-paper systems, which may originate from adhesives, coating binders, waxes, inks, and polymeric constituents [39]. Phthalate-containing materials have also been identified directly in chemically characterised stickies, together with acrylate- and vinyl-based polymers [40].

The co-enrichment of long-chain hydrocarbons, plasticisers, lipid-derived compounds, and other hydrophobic constituents suggests that the membrane-associated material may have contained adhesive-, coating-, wax-, or deposit-forming components capable of concentrating other non-cellulosic compounds during aqueous processing. Such behaviour is consistent with the tendency of stickies and related hydrophobic contaminants to associate with fibres, mineral particles, and other solid interfaces [39]. However, because the membrane-associated material was not independently identified as a specific sticky phase and equilibrium partition coefficients were not determined, the observed behaviour is more appropriately described as preferential association and chemical fractionation rather than adsorption or quantitative partitioning.

These findings are important for interpreting dissolution–regeneration as a purification step. A decrease in the concentration of a residual compound in the recovered RNC cannot by itself demonstrate its removal from the processing system. Instead, dissolution–regeneration followed by dialysis produced two chemically distinct recovered fractions, with 53.1% of the quantified residual organic load associated with the smaller membrane-associated fraction. No comparable fraction was observed when CH_3_COOH-pretreated cellulose underwent the same dissolution–regeneration procedure (Section 3.2.3), indicating that upstream feedstock purification affected not only the chemical residuum but also subsequent fractionation behaviour.

#### 3.2.2. Effect of Post-Regeneration Solvent Purification

Sequential post-regeneration purification with hexane and ethanol, followed by water washing, substantially simplified the residual non-cellulosic organic fingerprint of the recovered RNC (Figure 3a). After purification, waxy/polyolefinic hydrocarbons, aromatic and condensed aromatic compounds, lipid-derived, wax-like and oxygenated degradation products, terpenoid and biologically associated hydrophobic products, amide additives and aromatic amides, oxidised and secondary transformation products, and NIAS and polymer-derived transformation products included in the evaluated profile were no longer detected. Plasticisers and polymer additives decreased from 75.70 to 10.69 mg·kg^−1^, while process-related nitrogen- and sulphur-containing compounds decreased from 59.58 to 1.24 mg·kg^−1^. Thus, solvent purification markedly reduced both the concentration and chemical complexity of the residual fingerprint.

The effect was particularly evident for persistent long-chain hydrocarbons. Hexacosane, octacosane, nonacosane, and related higher *n*-alkanes detected before purification were no longer detected after treatment (Table S1), supporting their interpretation as extractable wax- or hydrocarbon-associated residues rather than cellulose-derived pyrolysis products. Such hydrocarbons in paper and cardboard may originate from mineral oil-containing materials, printing formulations, paraffinic waxes, coatings, and other hydrocarbon-based constituents [9,38].

Plasticiser-related compounds exhibited similarly pronounced but compound-selective changes. DEHP and bis(2-ethylhexyl) terephthalate (DEHT/DOTP) were no longer detected after purification, whereas dibutyl phthalate (DBP; 7.70 mg·kg^−1^) and acetyl tributyl citrate (ATBC; 2.99 mg·kg^−1^) remained detectable (Table S1). The residual fingerprint, therefore, contained both a phthalate ester and a non-phthalate alternative. Citrate esters and other non-phthalate plasticisers are increasingly used as alternatives to conventional phthalates in polymer and food-contact applications [41,42]. The different responses of individual plasticisers demonstrate that post-regeneration purification was compound-selective rather than exhaustive.

The observed changes are consistent with sequential extraction across different polarity ranges: hexane targets predominantly non-polar and strongly lipophilic constituents, whereas ethanol extends extraction toward more polar organic compounds. Combined polar/non-polar extraction has similarly been used to broaden the range of compounds recovered from recycled paper matrices [9]. But because the material was analysed only after completion of the entire hexane–ethanol sequence and subsequent water washing, the effects of the individual solvents cannot be distinguished, and the observed reduction must be attributed to the overall purification procedure.

ATR-FTIR provided no evidence of major modification of the cellulose backbone following solvent treatment, supporting the interpretation of the reduced fingerprint primarily as removal of extractable non-cellulosic constituents rather than chemical transformation of regenerated cellulose. Dissolution–regeneration and subsequent solvent purification, therefore, performed distinct functions: the former induced fractionation of residual paper-associated constituents, whereas the latter substantially reduced the extractable organic fraction remaining in RNC.

Despite this reduction, DBP, ATBC, and selected process-related nitrogen- and sulphur-containing compounds remained detectable. Moreover, solvent purification was applied downstream, after the formation of the contaminant-enriched membrane-associated fraction during dialysis. This prompted evaluation of an alternative process configuration in which the non-cellulosic burden was reduced before dissolution–regeneration by acetic acid pretreatment (Section 3.2.3).

#### 3.2.3. Effect of Acetic Acid Pretreatment Before Dissolution–Regeneration

A distinctly different residual organic profile was obtained when waste office paper was pretreated with 0.2 M acetic acid before dissolution–regeneration. Previous characterisation of the same feedstock demonstrated that this pretreatment efficiently removed the PCC-based mineral fraction and substantially reduced paper-associated organic constituents [11]. Consequently, dissolution–regeneration in this route was applied to an already substantially purified cellulose feedstock rather than directly to the original waste paper.

The summed concentration of the evaluated residual non-cellulosic compounds decreased from 38.21 mg·kg^−1^ in CH_3_COOH-pretreated cellulose to 20.01 mg·kg^−1^ in RNC-CH_3_COOH-Zhang et al. [20] (Route B), corresponding to a 47.6% decrease (Figure 3a). The response was strongly compound-dependent: triethyl citrate (TEC) decreased by 97.23%, octanoic acid by 86.81%, acetyl tributyl citrate (ATBC) by 80.23%, and 2-(2-hydroxyethoxy)ethyl octadecanoate by 69.63% (Table S1). Dissolution–regeneration of the pretreated cellulose, therefore, further reduced several plasticiser-related, lipid-derived, and process-related constituents remaining after acid purification.

The response was not uniform across all compounds. Most notably, DEHP increased from 4.72 to 5.64 mg·kg^−1^, while several minor compounds also exhibited higher concentrations after regeneration (Table S1). These increases cannot be interpreted as de novo formation because Py-GC/MS results are expressed relative to the analysed material, and no compound-specific mass balance was established for this processing route. Apparent increases may instead reflect preferential retention, differences in matrix composition, or concentration effects resulting from the removal of other material components. The results, therefore, demonstrate compound-selective modification of the organic residuum rather than uniform removal of non-cellulosic constituents.

A further technologically important difference was observed during dialysis. Direct dissolution–regeneration of untreated waste paper generated a membrane-associated fraction representing approximately 18% of the combined recovered dry mass but containing 53.1% of the quantified residual non-cellulosic organic load distributed between the two recovered solid fractions (Figure 3b). In contrast, no comparable membrane-associated fraction was observed after CH_3_COOH pretreatment. This suggests that this material formed from constituents retained in the untreated feedstock rather than as an inherent consequence of cellulose dissolution–regeneration. Upstream pretreatment, therefore, modified both the chemical burden entering the dissolution step and the subsequent fractionation behaviour during dialysis.

The final RNC products consequently differed markedly in their organic residuum: directly regenerated RNC contained 542.22 mg·kg^−1^, compared with 20.01 mg·kg^−1^ in RNC-CH_3_COOH-Zhang et al. [20] (Route B) (Figure 3a). Because the two routes involved different processing histories and the direct route additionally generated the chemically enriched membrane-associated fraction, this difference should not be interpreted as a direct process removal efficiency. Nevertheless, together with the previously demonstrated removal of PCC [11], the substantially lower residual concentration and absence of a comparable membrane-associated fraction demonstrate the combined effect of upstream feedstock purification.

The two purification strategies, therefore, performed different technological functions. Post-regeneration hexane–ethanol purification produced the lowest residual concentration (11.93 mg·kg^−1^), whereas upstream acetic acid pretreatment followed by dissolution–regeneration resulted in a 20.01 mg·kg^−1^ concentration while additionally removing PCC and preventing the formation of the membrane-associated fraction. Endpoint residual concentrations alone are, therefore, insufficient to rank the two strategies: the position of purification within the process influenced both the final chemical fingerprint and the fractionation behaviour of non-cellulosic constituents during subsequent processing.

#### 3.2.4. Comparison with Commercial CNCs as a Reference Material

Commercial CNCs were included as an independent reference to distinguish the intrinsic Py-GC/MS fingerprint of cellulose from residual non-cellulosic compounds and to compare waste paper-derived RNCs with a commercially produced nanocellulose material. As expected, its overall Py-GC/MS profile was dominated by cellulose-derived pyrolysis products, particularly anhydrosugars and other oxygenated carbohydrate degradation products. These compounds were excluded using the same criteria applied to the waste paper-derived materials; the comparison, therefore, reflects the evaluated residual non-cellulosic organic residuum rather than the total Py-GC/MS response.

**Figure 4:**
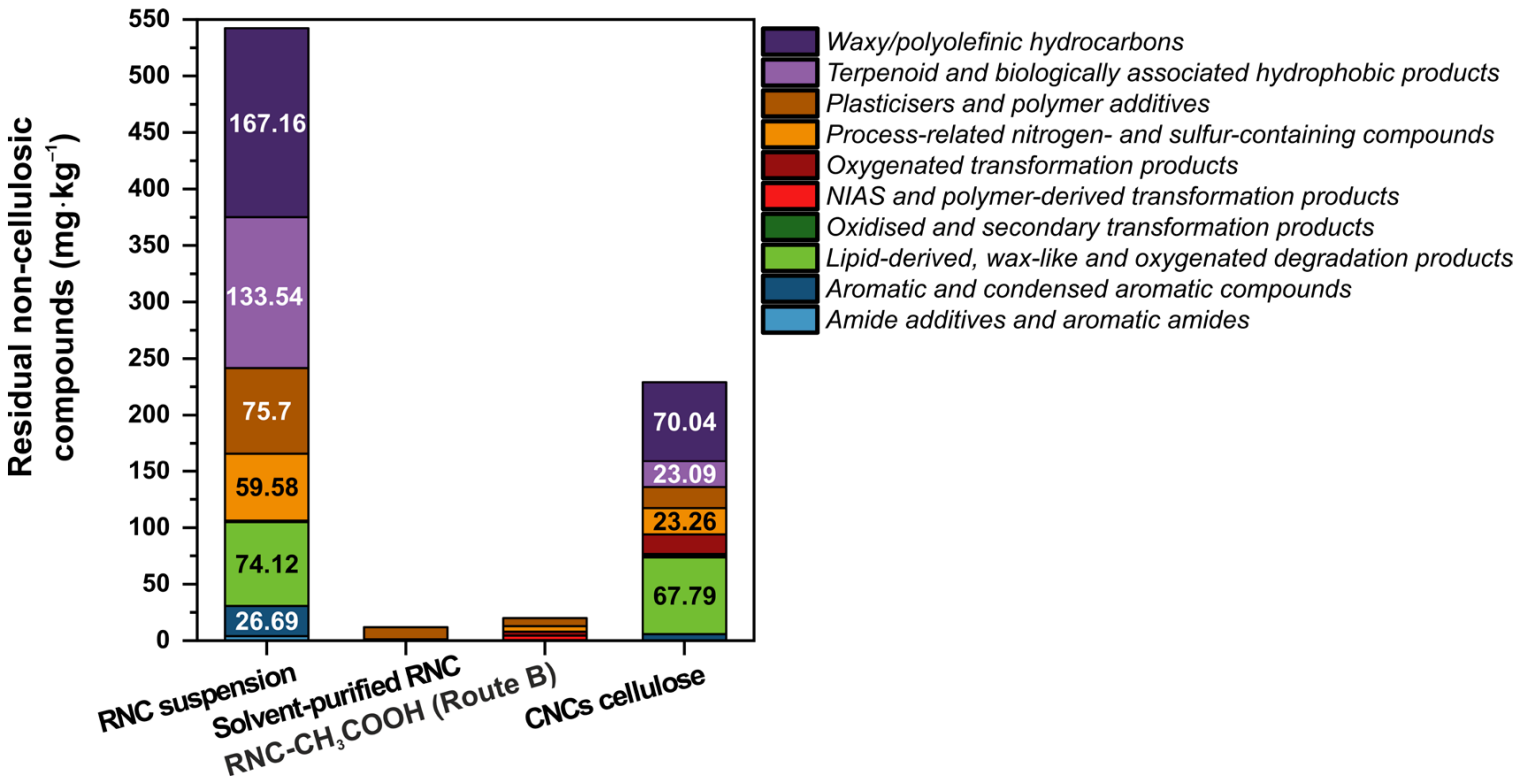
Sum of residual non-cellulosic organic compounds in waste paper-derived RNC materials and commercial CNCs determined by Py-GC/MS. Stacked bars represent summed concentrations of individual compound groups after exclusion of cellulose-derived pyrolysis products. Individual compounds are reported in Table S1.

Commercial CNCs nevertheless exhibited a measurable non-cellulosic organic residuum of 229.05 mg·kg^−1^, dominated by waxy/polyolefinic hydrocarbons (70.04 mg·kg^−1^), lipid-derived, wax-like and oxygenated degradation products (67.79 mg·kg^−1^), process-related nitrogen- and sulphur-containing compounds (23.26 mg·kg^−1^), terpenoid and biologically associated hydrophobic products (23.09 mg·kg^−1^), and plasticisers and polymer additives (18.62 mg·kg^−1^) (Figure 4). Long-chain hydrocarbons included octacosane, triacontane, heneicosane, and hexacosane, while DEHP, DEHT/DOTP, DBP, and ATBC occurred within the plasticiser-related fraction (Table S1). These constituents may reflect raw-material origin, manufacturing and purification history, or subsequent handling, and they demonstrate that a detectable organic residuum is not restricted to nanocellulose produced from recycled paper.

**Figure 5:**
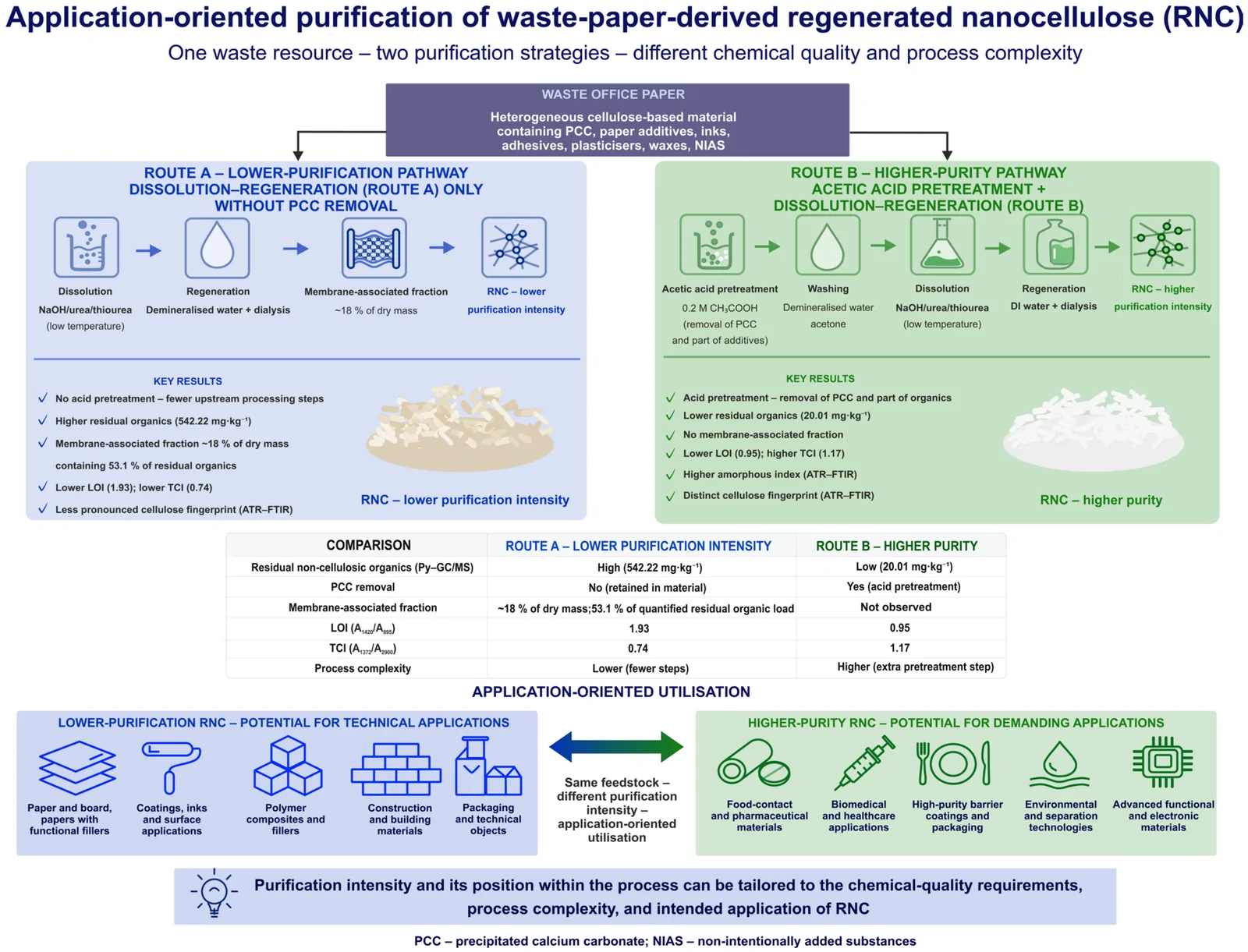
Application-oriented purification pathways for waste paper-derived regenerated nanocellulose (RNC). Route A (blue) represents direct dissolution–regeneration using the modified Zhang et al. [20] procedure without prior removal of precipitated calcium carbonate (PCC), whereas Route B (green) incorporates upstream acetic acid pretreatment based on Kucbel et al. [11] followed by the same dissolution–regeneration procedure. The scheme illustrates differences in the residual non-cellulosic organic fingerprint determined by Py-GC/MS, formation of the membrane-associated fraction, structural characteristics, process complexity, and potential application-oriented utilisation. Proposed applications are conceptual and require application-specific performance and safety assessment.

The final nanocellulosic materials differed markedly in both the magnitude and composition of their residual fingerprints (Figure 5). Directly regenerated RNC exhibited the highest concentration, whereas solvent-purified RNC (Route A) and RNC-CH_3_COOH-Zhang et al. [20] (Route B) contained only 11.93 and 20.01 mg·kg^−1^, respectively, compared with 229.05 mg·kg^−1^ in commercial CNCs. Thus, within the analytical framework applied here, both additionally purified waste paper-derived RNC materials exhibited substantially lower residual non-cellulosic organic residuum than the commercial reference.

A direct comparison with literature-reported residual organic contents requires caution because these values are defined and quantified using different analytical approaches. For comparison, the Py-GC/MS organic residuum corresponded to approximately 0.0012 wt.% for solvent-purified RNC, 0.0020 wt.% for RNC-CH_3_COOH-Zhang et al. [20] (Route B), and 0.0229 wt.% for commercial CNCs. These percentages represent only the sum of the quantified compounds comprising the residual organic fingerprint and cannot be interpreted as total impurity contents or converted directly into conventional material purity values. Ruiz-Caldas et al. [43], for example, reported organic impurity contents below 1% for most CNCs produced from postconsumer cotton and blended fabrics using solid-state 13C NMR. In contrast, Xu and Chen [44] reported approximately 99.99% CNC purity based on their analytical criteria following enzymatic treatment of pulp fibres. Purity and chemical composition are recognised as important characteristics of cellulose nanomaterials alongside morphology, surface chemistry, crystallinity, and thermal properties [45]. However, such bulk data are not directly comparable to the compound-resolved residual organic fingerprint determined in the present study by Py-GC/MS.

Accordingly, the lower organic residuum of the purified waste paper-derived RNC materials should not be interpreted as evidence that they are universally “purer” than commercial CNCs. Rather, the comparison demonstrates that the chemical legacy of recycled office paper is not necessarily retained in the final RNC and can be substantially reduced by appropriate upstream or downstream purification. More broadly, the results demonstrate that structural characterisation, bulk chemical purity assessment, and Py-GC/MS-based residual organic fingerprint analysis provide complementary information and should be considered together when evaluating nanocellulose derived from chemically complex secondary feedstocks [45].

### 3.3. How Low Should the Organic Residuum Be for Waste Paper-Derived RNC?

The results demonstrate that the selected purification strategy can substantially modify the chemical quality of waste paper-derived regenerated nanocellulose (RNC). Direct dissolution–regeneration retained a pronounced residual non-cellulosic organic residuum. It generated a membrane-associated fraction representing approximately 18% of the recovered dry mass but containing 53.1% of the quantified residual organic load. Additional purification substantially reduced this burden. These findings raise a broader technological question: should waste paper-derived RNC be purified to the maximum achievable level, or should purification intensity be tailored to its intended application?

Nanocellulose is being developed for applications ranging from paper, coatings, packaging, and polymer composites to food-contact materials, biomedical products, environmental technologies, and advanced functional materials [29,46]. These applications differ substantially in their requirements for chemical composition, extractable substances, safety, and material performance [47]. A single universal quality criterion is, therefore, unlikely to be appropriate for all nanocellulose applications. Structural quality, bulk chemical purity, and the residual organic fingerprint determined by Py-GC/MS represent related but distinct material characteristics that should be considered according to the intended application.

For applications involving direct or indirect human exposure, including food-contact, pharmaceutical, or biomedical uses, stringent control of residual chemical constituents is particularly important. Such applications require consideration not only of cellulose structure and morphology but also of extractable and leachable impurities and processing residues [32]. The detection of plasticisers, long-chain hydrocarbons, and other paper-associated constituents in some processing fractions illustrates why nanocellulose derived from chemically heterogeneous secondary feedstocks may require targeted purification for applications with stringent chemical-quality requirements.

Conversely, maximum purification may not be necessary for technical applications such as paper and board, barrier coatings, polymer composites, or construction-related materials [46,48]. In such cases, the benefit of additional purification should be weighed against chemical, solvent, water, and energy consumption, as well as potential material losses. Purification beyond that required for a defined application may increase resource demand, process complexity, costs, and environmental impacts without providing a corresponding functional benefit. Techno-economic and life-cycle assessments similarly emphasise the importance of chemical and energy inputs associated with pretreatment, purification, and recovery in determining the feasibility and environmental performance of nanocellulose production [15,16].

The present results further show that the position of purification within the process is an important consideration. Although post-regeneration hexane–ethanol treatment produced the lowest organic residuum (11.93 mg·kg^−1^), this downstream purification step is not directly comparable with upstream acetic acid pretreatment because the two approaches operate at different stages of the process and target different fractions of residual constituents. Acetic acid pretreatment removes mineral fillers and part of the non-cellulosic burden before dissolution–regeneration, whereas post-regeneration solvent purification removes extractable residual organic compounds from the regenerated material. Consequently, these approaches should be regarded as complementary rather than competing purification strategies. Upstream purification may additionally provide process benefits by modifying the behaviour of non-cellulosic constituents during subsequent processing, as evidenced by the absence of a comparable membrane-associated fraction after acetic acid pretreatment. Integrated fractionation concepts for lignocellulosic waste valorisation similarly emphasise consideration of both cellulose-product quality and the management or recovery of non-cellulosic fractions [2,49].

These findings support an application-oriented purification strategy for waste paper-derived RNC. More intensive or additional purification steps should be prioritised where residual constituents may compromise safety, regulatory compliance, or functional performance. In contrast, less intensive processing may suffice for technical applications where a higher organic residuum is acceptable.

Even after post-regeneration solvent purification, trace amounts of dibutyl phthalate (DBP) and acetyl tributyl citrate (ATBC) remained detectable. Although these residual concentrations were substantially lower than in untreated regenerated nanocellulose, their relevance ultimately depends on the intended application and the associated regulatory requirements. Applications involving direct human contact, food-contact materials, or biomedical applications may require a further reduction of these residual compounds, along with application-specific safety assessment. In contrast, such residual concentrations may be acceptable for many technical applications, subject to the relevant regulatory and performance requirements.

The present study does not establish application-specific acceptance limits or demonstrate suitability for individual end uses; these require application-specific testing. Rather, the results show that purification intensity and its position within the process can be deliberately selected according to the required chemical quality, structural properties, resource demand, process complexity, and intended application of the resulting RNC.

Although the structural analyses support the designation of regenerated nanocellulose, complementary nanoscale imaging techniques, such as transmission electron microscopy or atomic force microscopy, would further strengthen the morphological characterisation of the regenerated material and should be included in future studies.

The present study focused on structural reorganisation and the residual organic fingerprint determined by Py-GC/MS. Evaluation of additional physicochemical properties, including surface charge, morphology, mechanical performance, and thermal stability, was beyond the scope of this work and should be addressed in future studies, depending on the intended application of the regenerated nanocellulose.

## 4. Conclusions

Waste office paper can be successfully converted into regenerated nanocellulose, but the residual chemical quality of the resulting material strongly depends on the purification strategy. Direct dissolution–regeneration did not eliminate the non-cellulosic organic compounds and induced pronounced chemical fractionation: the membrane-associated fraction represented only 18% of the recovered dry mass but contained 53% of the quantified organic residuum recovered in the two solid fractions after dialysis. Dissolution–regeneration alone should, therefore, not be regarded as an inherently effective purification step.

Both additional purification strategies substantially reduced the organic residuum through different process routes. Post-regeneration hexane–ethanol purification, a downstream polishing step, produced the lowest organic residuum (11.9 mg·kg^−1^; ~0.0012 wt.%). In contrast, upstream acetic acid pretreatment followed by dissolution–regeneration resulted in 20.0 mg·kg^−1^ (~0.0020 wt.%) while additionally removing PCC and preventing formation of the membrane-associated fraction. Because these purification strategies operate at different stages of the process and fulfil different functions, they should be regarded as complementary rather than directly competing approaches. Both values were substantially lower than the quantified residual organic fingerprint determined for the commercial CNC reference (229.1 mg·kg^−1^; ~0.0229 wt.%). These percentages represent only the sum of the quantified compounds comprising the residual organic fingerprint determined by Py-GC/MS. They should not be interpreted as conventional bulk impurity contents or overall material purity. Nevertheless, the results demonstrate that the chemical legacy of recycled paper is not necessarily retained in the final regenerated nanocellulosic material.

These findings support an application-oriented purification strategy for nanocellulose derived from secondary raw materials. Upstream acetic acid pretreatment appears particularly advantageous as a baseline purification step because it simultaneously reduces the mineral and organic burden before dissolution–regeneration and prevents formation of the membrane-associated fraction. Post-regeneration solvent purification can further reduce the residual organic fingerprint when exceptionally low residual concentrations are required. Rather than pursuing a single universal purification target, purification intensity should be selected according to the intended application, considering structural properties, residual organic fingerprint, material yield, process complexity, and resource consumption.

Although the present study focused on comparing the effectiveness of different purification strategies in reducing the organic residuum, practical implementation will additionally require evaluation of solvent consumption, solvent recovery efficiency, energy demand, and associated material losses. These process-engineering aspects were beyond the scope of the present work. Still, they should be considered in future studies to assess the overall sustainability and industrial feasibility of the proposed purification strategies. Waste-paper valorisation can thus be designed to produce regenerated nanocellulose with a residual organic fingerprint and structural properties tailored to the requirements of its intended application.

## Figures and Tables

**Table 1:** XRD-derived crystallinity index (CrI) and predominant crystal structure of the investigated cellulose materials.

Material	Predominant Crystal Structure	CrI (%)
Commercial CNC (Nanografi)	Cellulose I	70.6
Regenerated nanocellulose (RNC–CH_3_COOH–Zhang et al. [20]; Route B)	Cellulose II-like	61.6

Explanation: Based on the XRD pattern, regenerated nanocellulose exhibited diffraction features characteristic of Cellulose II. However, quantitative phase analysis was not performed; therefore, the designation “Cellulose II-like” indicates the development of structural features characteristic of Cellulose II rather than complete polymorphic transformation.

**Table 2:** Characteristic ATR-FTIR absorption bands observed in regenerated nanocellulose samples and the commercial cellulose nanocrystal (CNC) reference materials.

Wavenumber (cm^−1^)	Band Assignment	Commercial CNCs	RNC—Zhang et al. [20] (Solvent-Purified)	RNC—Zhang et al. [20] (Route A)	RNC—CH_3_COOH–Zhang et al. [20] (Route B)
3340–3330	O–H stretching (hydrogen-bonded hydroxyl groups)	3339.95	weak/unresolved	3327.78	3329.55
2925–2850	C–H stretching (aliphatic C–H groups)	2887.60	2924.28; 2853.98	2893.71	2887.65
1640–1630	H–O–H bending (adsorbed water)	weak/unresolved	unresolved	unresolved	1630.80
1430–1420	CH_2_ scissoring vibration	1426.66	unresolved	1423.60	weak/unresolved
1370–1310	C–H bending/O–H in-plane bending	unresolved	unresolved	1316.39	unresolved
1161–1160	Asymmetric C–O–C stretching	1160.75	unresolved	1161.15	1160.77
1090–986	C–O stretching/pyranose ring vibrations	1088.85; 1026.27	unresolved	1056.72; 1030.35	1037.11; 986.55
898–891	β-Glycosidic linkage	891.79	unresolved	897.90	891.80
668–534	Low-frequency bands (limited diagnostic value)	662.56	—	572.21; 563.39; 534.63	668.56; 556.43
2359–2340	Atmospheric CO_2_ (instrumental artefact; not interpreted as a sample band)	—	—	—	2359.66

Note: Weak/unresolved indicates that the corresponding absorption band was either absent or insufficiently resolved for a reliable assignment.

**Table 3:** FTIR-derived structural indices of commercial CNCs and waste paper-derived regenerated nanocellulose.

Sample	LOI (A_1420_/A_895_)	TCI (A_1372_/A_2900_)
Commercial CNCs (Nanografi)	2.29	0.40
RNC-Zhang et al. [20] (Route A)	1.93	0.74
RNC-Zhang et al. [20]-solvent	1.50	0.53
RNC-CH_3_COOH-Zhang et al. [20] (Route B)	0.95	1.17

Note: LOI, lateral order index; TCI, total crystallinity index. Both parameters are empirical FTIR-derived spectral ratios and are interpreted as complementary indicators of changes in cellulose molecular organisation rather than as direct quantitative measures of crystallinity.

**Table 4:** Dry matter concentration and recovered dry mass of regenerated nanocellulose prepared from waste office paper before and after acetic acid pretreatment.

Parameter	RNC-Zhang et al. [20] (Route A)	RNC-CH_3_COOH-Zhang et al. [20] (Route B)
Dry matter concentration (g·L^−1^)	13.7	10.0
Total dry mass recovered from 250 mL (g)	3.43	2.50
Equivalent production (g·kg^−1^ waste office paper)	685	500

## Data Availability

The original data presented in the study are openly available in the Zenodo repository at https://doi.org/10.5281/zenodo.21990276 (accessed on 18 August 2026).

## Supplementary Materials

The following supporting information can be downloaded at: https://www.mdpi.com/article/10.3390/polym18182242/s1, Table S1: Residual non-cellulosic compounds identified by Py-GC/MS across nanocellulose processing routes.

## Abbreviations

The following abbreviations are used in this manuscript:

ATBC	Acetyl tributyl citrate
ATR	Attenuated total reflectance
ATR-FTIR	Attenuated total reflectance Fourier-transform infrared spectroscopy
CNC	Cellulose nanocrystal
CNFs	Cellulose nanofibers
DBP	Dibutyl phthalate
DEHP	Bis(2-ethylhexyl) phthalate
DEHT/DOTP	Bis(2-ethylhexyl) terephthalate, commonly referred to as dioctyl terephthalate
FTIR	Fourier-transform infrared spectroscopy
GC	Gas chromatography
GC/MS	Gas chromatography/mass spectrometry
IUPAC	International Union of Pure and Applied Chemistry
LOI	Lateral order index
NIAS	Non-intentionally added substances
NIST	National Institute of Standards and Technology
NMR	Nuclear magnetic resonance
PCC	Precipitated calcium carbonate
Py-GC/MS	Pyrolysis–gas chromatography/mass spectrometry
RNC	Regenerated nanocellulose
TCI	Total crystallinity index
TEC	Triethyl citrate
XRD	X-ray diffraction
